# Engaging Equity Pedagogies in Computer Science Learning Environments

**DOI:** 10.26716/jcsi.2020.03.2.1

**Published:** 2020-11-25

**Authors:** Tia C. Madkins, Nicol R. Howard, Natalie Freed

**Affiliations:** The University of Texas at Austin; University of Redlands; The University of Texas at Austin

**Keywords:** equity, equity-focused pedagogies, culturally relevant computing

## Abstract

In this position paper, we advocate for the use of equity-focused teaching and learning as an essential practice within computer science classrooms. We provide an overview of the theoretical underpinnings of various *equity pedagogies* (Banks & Banks, 1995), such as *culturally relevant pedagogy* (Ladson-Billings, 1995, 2006) and share how they have been utilized in CS classrooms. First, we provide a brief history of CS education and issues of equity within public schools in the United States. In sharing our definition of equity, along with our rationale for how and why these strategies can be taken up in computer science (CS) learning environments, we demonstrate how researchers and educators can shift the focus from access and achievement to social justice. After explaining the differences between the relevant theoretical frameworks, we provide practical examples from research of how both practitioners and researchers might use and/or examine equity-focused teaching practices. Resources for further learning are also included.

Prior reviews related to computational thinking and the state of the field (e.g., Grover & Pea, 2013; Wing, 2006) have outlined distinctions between important ideas, orientations to the work, and broadening the scope of the discourse. Additional research has defined computational thinking and computer science, addressed the state of the field, and offered guidance on exploring the relationship between computational thinking and various content areas (Grover & Pea, 2013; Heitin, 2015; Jacob & Warschauer, 2018). As the state of the field changes, educational institutions are shifting how they prepare students for the field and more attention is given to how teachers deliver learning experiences (N. Howard, 2019). Similar to other researchers, we hope to provide an understanding of the current state of the field related to computer science (CS) education in the United States and offer guidance on situating equity pedagogies (e.g., *culturally relevant pedagogy*, *culturally relevant computing*) as an essential practice. Therefore, in this position paper, we argue that both researchers and practitioners need to understand and engage equity pedagogies in computer science learning environments. To this end, we offer guidance on 1) the distinctions in frameworks that provide the theoretical underpinnings of commonly used equity pedagogies and 2) how researchers and educators might engage in investigating and utilizing these teaching practices.

First, we provide an overview of the state of the field of K-12 CS education in the U.S. and share our working definition of equity. Then, we explain four theoretical frameworks undergirding commonly used equity pedagogies in K-12 settings: *culturally relevant pedagogy* (CRP; Ladson-Billings, 1995, 2006); *culturally relevant teaching* (CRT; Gay, 2000); *culturally sustaining pedagogy* (CSP; Paris & Alim, 2012); and *culturally responsive computing* (CRC; K. Scott et al., 2013, 2015). Next, we highlight prior research efforts demonstrating how equity pedagogies have been taken up in CS learning environments and provide practical examples of these strategies in K-12 classrooms. Finally, we provide a resources list for further learning in this area. In doing so, we hope to provide guidance to both practitioners and researchers for taking up the ideas we present in this position paper.

## State of the Field: K-12 CS Education and Relationship to CS for All

The last 15 years have seen a renewal of interest in K-12 computer science education, along with a new effort to define its goals and questions about how best to reach them (Grover & Pea, 2013). Computer scientist Jeanette Wing’s (2006) call to action helped the discussion begin to coalesce around an organizing principle: computational thinking. While this term was not new (Caeli & Yadav, 2020; Papert, 1980), Wing revived it in the context of 21^st^ century challenges. She argued that beyond just using computer programs or learning to code, the ability to “think like a computer scientist” was a fundamental skill relevant to all students (Wing, 2006, p. 35).

The next few years saw a number of new initiatives aimed at assessing and improving the state of K-12 CS education. In 2010, a report conducted by the Association for Computing Machinery (ACM) and the Computer Science Teachers Association (CSTA) identified three gaps in K-12 CS education: a lack of cohesive standards, discrepancies between states, and a gap in access and participation by race, gender, and socioeconomic groups, especially evidenced in the low rates of women and minoritized students taking the Advanced Placement (AP) CS exam (Wilson et al., 2010). The *AP Computer Science Principles Framework* was later released, establishing a new Computer Science AP course in 2017. The new course, *AP CS Principles*, was designed with the explicit goal of engaging minoritized students, in direct response to the nationwide participation gap in high school level computer science (Cuny, 2012; desJardins, 2015).

Influenced by these efforts, the Obama Administration announced the Computer Science for All Initiative in January 2016. This initiative allocated several billions of dollars in funding to expand access to K-12 computer science across the United States. Another outcome of the initiative was the founding of the CS for All Consortium, whose initial members included school districts, content providers, local and national organizations, nonprofits such as Code.org, and tech companies. Additionally, computing leaders and organizations like ACM, Code.org, and CSTA joined forces to develop a framework to define K-12 CS education. The collective work of CSTA, the International Society for Technology in Education (ISTE), and other organizations culminated in the *K-12 Computer Science Framework* and a set of aligned standards (CSTA standards, ISTE standards). Each set of standards addresses equity, specifically related to students’ access to learning opportunities and achievement in inclusive classroom cultures. For example, the ISTE Computational Thinking Competencies include an Equity Leader strand to give educators a framework whereby they can set goals for teaching and learning. The Equity Leader strand explicitly calls for CS educators to counter stereotypes that exclude any students from CS opportunities; it sets the expectation that educators choose culturally relevant learning activities (ISTE, 2016, 2017). The CSTA Standards for Computer Science Teachers also include an Equity and Inclusion domain that addresses a set of CS practices that support teachers with sharpening their equity-focused vision (CSTA, 2020). Under this domain, CSTA notes that effective CS teachers are to examine issues of equity and consciously work towards “an intentional, equity-focused vision to improve access, engagement, and achievement” for all students (CSTA, 2020, Standard 2, p. 2).

The inclusion of computer science in elementary classrooms has become more prevalent over the past ten years as well, especially since the launch of new programs and initiatives such as Girls Who Code and Code.org (N. Howard & K. Howard, 2020; A. Scott et al., 2016). Prior research has indicated girls’ early exposure to CS results in the same level of interest, confidence, motivation, and high school course-taking efforts as boys (Master et al., 2017). Although more high schools are including CS as a core class, girls and racially and ethnically minoritized students are still largely absent from the 45% of our nation’s high schools that teach CS (Code.org, 2019). Questions remain as to why fewer girls and minoritized students are represented in CS courses. Additionally, there is the question of whether fast-growing initiatives and low levels of access impact the quality of CS content development and delivery. More specifically, one question is—Are equity-focused pedagogies taken up effectively in all CS learning environments? Thus, we now turn our attention to defining equity and then discuss why and how we should use equity-focused pedagogies in CS.

### Equity defined.

For some time, STEM education scholars have offered perspectives on how we should define *equity* and how equity-focused initiatives might be taken up in both research and practice based on these definitions (e.g., Secada, 1989; R. Gutiérrez, 2002; Philip & Azevedo, 2017; Tate, 1995). In educational research, it is common for educators and researchers to define *equity* similarly to *equality*, with a focus on fairness and equal access to inputs, such as resources (i.e., technology, textbooks, classroom spaces), advanced course offerings, highly-qualified teachers, and standards-based instruction (O. Espinoza, 2007; R. Gutiérrez, 2008; Madkins, 2016). Others explore equity as related to equitable student outcomes, such that their work focuses on disparities in student achievement measures, like NAEP scores, degree attainment, broadening participation efforts (e.g., Obama CS for All Initiative; NAEP, 2016; NRC, 2012). In contrast, scholars who have critical stances grounded in social justice call out and seek to address sociopolitical influences on society and schooling (e.g., Bang et al., 2017; M. Espinoza et al., 2020; R. Gutiérrez, 2009; Windschitl & Calabrese Barton, 2016; Vakil, 2018). Collectively, these scholars define equity in ways that acknowledge minoritized students’ full humanity and view their cultural, linguistic, and other practices as assets and resources for learning rather than deficits (i.e., assets-based rather than deficit-oriented approaches^1^). Working towards equity means supporting minoritized students in: 1) engaging in meaningful and rigorous instruction; 2) grappling with and challenging systemic racism, power, and oppression; and 3) using STEM and CS to empower themselves and their communities. As such, *equity* is defined as intentionally facilitating justice-oriented learning experiences for minoritized students. This requires viewing teaching and learning as *inseparable* from pursuing justice while attending to students’ access to rigorous instruction and equitable outcomes. Our research and arguments in this position paper are aligned with this definition of equity.

## Equity Pedagogies in STEM and CS Education: If Not Now, When?

Minoritized students have historically been marginalized in K-12 learning environments and consistently experience gaps in access to rigorous learning opportunities (i.e., opportunity gaps) in US public schools (Carter & Welner, 2013; Williams et al., 2014). Educational research is rife with descriptions of minoritized students’ disparate access to rigorous instruction and disparate achievement outcomes (Chakrabarti et al., 2019; Sargrad et al., 2019), especially in STEM education (NRC, 2012). For example, we know that Black and Latinx students are often denied access to gatekeeper mathematics courses (i.e., eighth grade Algebra) that influence their secondary and post-secondary STEM trajectories (Morton & Riegle-Crumb, 2019). We also know that many minoritized students uniquely experience these opportunity gaps as STEM majors (Riegle-Crumb et al., 2019) and contend with racism in pursuit of undergraduate and graduate STEM degrees (Ortiz et al., 2019; Watkins & Mensah, 2019). In CS education specifically, inequities are pervasive. This includes students’ access to technology, K-5 learning opportunities, advanced course offerings (e.g., AP courses), and highly-qualified teachers who utilize effective teaching strategies (Brannon & Novak, 2019; CSforAll, 2020; K. Howard & Havard, 2019; N. Howard & K. Howard, 2020; Madkins et al., 2019; Margolis & Goode, 2016).

In order to elucidate how teachers might better support minoritized students and decrease opportunity gaps, several scholars introduced theoretical frameworks based on their research. This body of research that began in the 1990s initially gained traction across educational research and practitioner communities as part of multicultural education efforts (Banks, 2006; Sleeter, 2012) and/or preparing to teach diverse student populations (Cochran-Smith, 2003). The pedagogical approaches grounded in these theoretical frameworks can be classified as *equity pedagogies*. This term, *equity pedagogies*, collectively describes assets-based pedagogical approaches that support minoritized students’ learning outcomes and further develop their potential^2^ to become social change agents (C. Banks & J. Banks, 1995). Banks and Banks (1995, p. 152) point out “it is not sufficient to help students learn to read, write, and compute within the dominant canon without learning also to question its assumptions, paradigms, and hegemonic characteristics.” Thus, using any equity pedagogy with fidelity requires teachers to simultaneously focus on learning *and* critical consciousness development^3^ [i.e., one’s familiarity with, recognition of, and desire to act upon inequities within sociohistorical and sociopolitical context (Freire, 1970/2005; Gay & Kirkland, 2003; Ladson-Billings, 1995)].

### Using equity pedagogies.

There is considerable evidence that using equity pedagogies can support minoritized students’ learning outcomes (i.e., development of conceptual knowledge, achievement, and identity development) across all content areas (Allen-Handy et al., 2020; Boaler & Sengupta-Irving, 2016; Allen et al., 2012; Tsurusaki et al., 2013). Much of this research has been conducted in English language arts (ELA; e.g., Duncan-Andrade, 2007), social studies (e.g., Stovall, 2006), early childhood education (e.g., Souto-Manning & Martell, 2017), or bilingual education (e.g., Irizarry & Antrop-González, 2007). Collectively, this research demonstrates how educators might simultaneously further develop students’ critical consciousness and support learning goals.

Scholars outside of STEM education have demonstrated the potential of using equity pedagogies with minoritized youth for some time. Research suggests this is because educators often find it easier to connect social justice issues to ELA and social studies curricula and content than to STEM content (Ladson-Billings, 2006; Sleeter, 2012; Young, 2010). However, scholars in mathematics and science education were among the first STEM education researchers to pursue this line of research (e.g., Bryan & Atwater, 2002; Calabrese Barton, 2003; R. Gutiérrez, 2002; Hines, 2003; Secada, 1989; Tate, 1995). Building on this initial work, scholars with justice-centered approaches to STEM teaching and learning advocate for addressing issues of equity and/or investigating equity pedagogies within teacher education and K-12 STEM classrooms. For example, Martin and colleagues have long investigated issues of equity and the hierarchical relationship between Black students, mathematics, and mathematics education (Gholson & D. Martin, 2014, 2019; Leonard & D. Martin, 2013; D. Martin, 2003, 2009, 2019). Others have conducted literature reviews that elucidate the variation in equity pedagogies and/or have explored how and why STEM education scholars and educators might take them up in research and practice (e.g., Boaler & Sengupta-Irving, 2016; Brown, 2017; Castaneda & Mejia, 2018; Mensah & Larson, 2017). All of this scholarship points to the importance of addressing long standing issues of systemic racism, power, and exclusionary practices in STEM education and fields. In so doing, we work towards creating and sustaining more just and equitable educational futures for all students, especially minoritized students, in our current sociopolitical climate (Bang, 2020; Sengupta-Irving & Vossoughi, 2019; Vakil & Ayers, 2019).

### Equity and CS in our current climate.

Given our current sociopolitical climate, it is important to engage equity pedagogies across content areas in K-12 learning environments, especially in CS classrooms. It is undeniable that our global public health (i.e., COVID-19 pandemic), race, and education crises are inextricably linked as evidenced by numerous events during Summer 2020. In the COVID era, we have witnessed: the exacerbation of educational inequalities—particularly with students’ access to technology and Internet at home; more frequent and highly publicized occurrences of police brutality and murders; and increased political divisiveness. As such, many Americans have increased the amount of time they spend online (Koeze & Popper, 2020) and cloud computing usage (Tozzi, 2020). Inevitably, this has increased attention to the applications and integration of computer science and other fields (e.g., Mitchum, 2020), such as artificial intelligence, data science, and social computing. In particular, social media activism has increased and highlighted how CS knowledge and skills, tech tools, and issues of equity intersect. Hashtags such as #ShutDownSTEM and #ShutDownAcademia brought attention to the intersectional experiences of individuals from minoritized communities in STEM, academia, and beyond both nationally and globally. This effort, held June 10, 2020 worldwide, was initiated by a group of researchers and academics to bring scientific, academic, and other communities together to “transition into a lifelong commitment of actions to eradicate anti-Black racism in academia and STEM” (see https://www.shutdownstem.com/ for more information). These sentiments are echoed in both popular press and scholarly work documenting the experiences of professionals in computer science and tech-related higher education and fields (e.g., Gilpin, 2015; Rankin & Thomas, 2020; A. Scott et al., 2017).

Relatedly, there has also been increased attention to understanding and addressing issues of equity in CS education, CS, and tech fields. For example, the CS/tech diversity problem (i.e., the need to broaden participation of individuals from racialized, gendered, and other minoritized communities) is well-documented in both K-20 education and the U.S. workforce (Gaskins, 2016; NSB, 2020; Ong et al., 2011; Washington, 2020). Broadening participation efforts to diversify the computing workforce have included the National Science Foundation’s (NSF) Broadening Participation in Computing Alliance Program, coding boot camps (e.g., Code Mississippi), and initiatives and research focused on Historically Black Colleges and Universities (HBCUs; see NSF HBCU Undergraduate Program). There have been several in and out of school education initiatives to broaden participation, such as CSforAll, *Exploring Computer Science* (ECS), and Black Girls Code (CSforAll, 2020; Goode & Margolis, 2011; see A. Scott et al., 2016 for a full review). Some programs, such as ECS, were launched with an explicit focus on broadening access to computer science and addressing issues of equity (Goode & Margolis, 2011; Margolis et al., 2008). Tech companies also continue to engage in philanthropy efforts to increase K-20 students’ access to technology and CS/tech education (e.g., Apple and ConnectED Initiative, Dell Foundation, Google Education, IBM Quantum Education and Research Initiative for Historically Black Colleges and Universities).

Though broadening participation efforts are important for achieving equal access *and* diversity goals, *we must also* move beyond participation narratives to address pervasive inequities, racism, and racist practices within CS education and the computing, tech, and related fields. In doing so, we not only work towards equity and inclusion, but can also empower students to integrate their computer science knowledge with efforts to solve issues relevant to minoritized communities. This might include addressing the well-documented racism and sexism in many algorithms, apps, and tech solutions (Akhtar, 2019; Benjamin, 2019; Braithwaite, 2020; Buranyi, 2017; Diente, 2020; Guynn, 2015; Madkins, 2016; Pinkerton, 2016; Resnick, 2019). *All* students must be explicitly taught to *recognize*, *examine and challenge* narratives and/or their own beliefs that algorithms *cannot* be racist, and *use* their computing knowledge and skills to uplift minoritized communities (Benjamin, 2019; Cheney-Lippold, 2017; Madkins, 2016).

There are several notable moves in this direction across CS educational research and practitioner communities. Professional associations have established special interest groups, conferences, and fellowships dedicated to equity-focused teaching and research (e.g., RESPECT, CSTA Equity Fellows). Additionally, researchers have focused on addressing issues of equity related to CS teaching and learning in special issues of scholarly journals and/or reports (e.g., Garcia & Morrell, 2013; Microsoft, 2019; K. Scott & Clark, 2013). Educator and scholar activists, scholars, and others have also called out racism, sexism, and antiblackness and/or made commitments to addressing equity within K-20 CS education (e.g., Benjamin, 2016, 2019; Goode et al., 2020; Guzdial, 2020a/b; Morales-Doyle et al., 2020; Payton et al., 2020; Sherriff et al., 2020; Washington, 2020; White, 2017). To support researchers and educators in doing *this* kind of equity-focused work, we now focus our attention on four theoretical frameworks that undergird commonly used equity-focused teaching practices in K-12 CS learning environments. In the next section, we provide an overview of these theoretical perspectives followed by a discussion of each framework.

## Equity Pedagogies: Differentiating the Theoretical Frameworks

We now turn to a discussion of four theoretical frameworks that undergird some of the equity-focused teaching practices teachers might engage in CS classrooms: *culturally relevant pedagogy* (CRP; Ladson-Billings, 1995, 2006); *culturally responsive teaching* (CRT; Gay, 2000); *culturally sustaining pedagogy* (CSP; Paris & Alim, 2014); and *culturally responsive computing* (CRC; K. Scott et al., 2013, 2015). There are other scholars whose theoretical frameworks contribute to our understandings and implementation of facilitating equitable and rigorous CS learning opportunities for minoritized students. For example, C. H. Lee and Soep (2016, p. 484) introduced *critical computational literacy*, which connects computational thinking (Wing, 2006) and critical literacy (“observing, analyzing, and deconstructing” oppressive systems and inequalities). However, we choose to highlight the following four frameworks because they provide the theoretical foundation for much of the current research and practice across learning contexts based on our review of the extant literature. These assets-based approaches specifically aim to support minoritized students across content areas, and a more comprehensive review of frameworks and related research is outside the scope of this manuscript (for reviews, see Aronson & Laughter, 2016; Morales-Chicas et al., 2019; Vakil, 2018). In Table 1, we provide an overview of the four frameworks we highlight in this paper, which is followed by a detailed discussion of each framework.

### Culturally Relevant Pedagogy (CRP).

*Culturally relevant pedagogy* (CRP) is a pedagogical strategy that can be utilized by teachers to effectively teach students from racially, ethnically, and linguistically minoritized communities and promotes equitable instruction (T. Howard, 2001; Ladson-Billings, 2012). Proper use of culturally relevant teaching in the classroom demonstrates a teacher’s deliberate and explicit acknowledgment that they value all students in their classroom and expect students to excel. The main tenets of CRP are (a) all students need to have academic success (both achievement and learning), so teachers must have high expectations for all students; (b) teachers support students’ development and maintenance of cultural competence by utilizing students’ culture for learning and helping them develop cultural capital to succeed in the dominant culture; and (c) teachers plan and facilitate learning experiences to further develop students’ critical consciousness, including awareness of societal and schooling inequities and challenging the status quo (Ladson-Billings, 1995, 2012).

Ladson-Billings (2006, 2012) emphasizes the importance of *each* aspect of CRP, noting that teachers must give equal attention to each component to implement culturally relevant teaching with fidelity. Furthermore, there is no *one particular way* that teachers should use CRP in their classrooms. Rather, the emphasis is on how the teacher thinks about their students (Ladson-Billings, 2006) and each teacher will have their own version of how the theory is enacted in the classroom (Abt-Perkins, 2011). The culturally relevant teacher recognizes they are preparing students to think critically and be “highly competent” in order to deal with inequities in our world *and* moves from feeling *sympathy for* her students to being *empathetic with* them (Ladson-Billings, 2006, p. 30). They also show their students they genuinely care for them (Ladson-Billings, 2006), reject deficit-model thinking about minoritized students (T. Howard, 2003, 2012; Ladson-Billings, 2006), and view students’ cultural practices as resources for learning (T. Howard, 2003).

### Culturally Responsive Teaching (CRT).

Geneva Gay (2000) defines culturally responsive teaching as situating the lived cultural experiences, characteristics, and perspectives of ethnically diverse students as a primary channel to inform effective teaching. Culturally relevant pedagogy refers to the theoretical framework whereas culturally relevant teaching refers to teaching practices that are grounded in the theory (Ladson-Billings, 2006; Milner, 2011). Some researchers also refer to this as culturally responsive teaching (Gay, 2000). The five essential elements of culturally responsive teaching include the following: (a) developing a cultural diversity knowledge base; (b) designing culturally relevant curricula; (c) demonstrating cultural care and building learning communities; (d) effective cross-cultural communications; and (e) cultural congruity.

To develop explicit knowledge about cultural diversity, educators need to understand different cultural characteristics and the contributions of various ethnic groups through the acquisition of factual information (Gay, 2000). Once this knowledge is acquired, teachers transfer it into the formal design of culturally relevant curricula. Prior to the delivery of instruction, teachers then create a classroom climate of cultural care in partnership with their students that is conducive to learning, while fostering communal learning environments. These learning communities should be inclusive of cross-cultural communications. Teachers also prioritize learning the communication styles of their students to avoid violating cultural values and to achieve effective cross-cultural communication in classrooms. Along with understanding communication styles, teachers establish cultural congruity by taking everything they know about their students’ cultural diversity (and how their students learn) to enhance their instructional delivery.

Zaretta Hammond (2015a/b) builds on the five essential elements provided by Gay (2000) through her work on culturally responsive teaching and the brain by identifying four practice areas: awareness, learning partnerships, information processing, and community building. Hammond asserts that culturally responsive teachers develop their own sociopolitical consciousness and possess an awareness of how systems award privileges to some and not to others. A culturally responsive teacher should have an awareness of how their own lens of culture affects their individual and collective views of students *and* how these views impact their instructional practices. Building a learning partnership with students in the process requires teachers to move away from didactic approaches to reimagine the relationship as a partnership for education. Connecting learning partnerships with authentic opportunities to make cultural connections allows teachers to become the “conduit that helps students process what they are learning” (Hammond, 2015b, p. 19). The culturally responsive teacher works to create an environment where minoritized students can take risks because they feel they belong and are fully supported.

### Culturally Sustaining Pedagogy (CSP).

Building upon culturally relevant pedagogy (CRP; Ladson-Billings, 1995), Paris and Alim (2012, 2014, 2017) conceptualized *culturally sustaining pedagogy* (CSP). This theoretical approach builds upon and seeks to promote equity and access via engaging minoritized students in learning experiences that sustain cultural pluralism. Undergirding this approach are beliefs that educators must recognize and build upon the varied and dynamic nature of students’ repertoires of practice (K. Gutiérrez & Rogoff, 2003). Moreover, educators must reject the idea that learning to navigate the dominant culture is the *only* way for students to be successful. In so doing, students have opportunities to learn that can sustain their cultural ways of being but also shape their access to power in oppressive systems—especially in schools and urban areas where they are the majority student population (Paris & Alim, 2017). By utilizing CSP, educators and students alike seek to understand, explore, and critique the ways in which racialized, ethnic, and/or cultural experiences (e.g., what it means to be Latinx or Black in a particular context) are rooted in both enduring *and* shifting practices. Paris and Alim (2017) urge educators and students to examine the ways that *hip hop pedagogy*, for example, may have undertones of liberation in some cases, yet reify hegemonic discourses in others (see Cummings et al., 2019 for a review of hip hop pedagogy in STEM education). By exploring the complexities of students’ cultural practices, teachers and students can engage in multidimensional and robust learning (C. D. Lee, 2017).

### Culturally Responsive Computing (CRC).

Guided by the rich, extensive research on culturally relevant teaching (Gay, 2000; Ladson-Billings, 1995, 2006), Kimberly Scott and colleagues (2013, 2015) introduced *culturally relevant computing* (CRC). The framework was initially developed to make connections between culturally relevant teaching practices and how “to make technologies and technology education accessible to diverse sociocultural groups using asset building approaches.” (K. Scott et al., 2015, p. 413). These principles support broadening participation efforts that focus on increasing K-12 students’ access to computing and diversity in the tech fields, but with a focus on the inextricable link between STEM + computing content and social justice (K. Scott et al., 2015).

CRC embeds sociocultural relevance at all levels of the learning experience, from the selection of tools and the actual learning environment to applications outside of the original learning context. Thus, students are supported to creatively engage with technology in a meaningful context with attention to their cultural and personal interests while addressing intersectional identities that impact their experiences with technology. CRC calls for situating technology ideas within a sociopolitical context and giving students opportunities to critique and explore issues that are relevant to them. Students should feel supported in their learning, identity development, and expression as they become creative innovators with technology, able to repurpose computing tools towards their own goals. When taking up CRC, educators redefine “success” of computing programs by asking “who creates, for whom, and to what ends,” rather than gauging success by a list of CS topics covered or on what technologies students can access (K. Scott et al., 2015, p. 421).

In summary, CRC principles include the following: 1) developing technologies that reflect and respond to minoritized students’ identities; 2) critiquing and addressing sociopolitical issues within computing and society more broadly; and 3) facilitating computing learning experiences that are grounded in and build upon a rigorous curriculum *and* minoritized students’ identities (e.g., academic, racialized, gendered) and cultural and linguistic practices (Eglash et al., 2013; K. Scott, et al., 2015).

## Equity Pedagogies in CS education: An Overview of Prior Research Efforts

In this section we provide a brief summary of the research literature related to student outcomes and the use of equity pedagogies in computer science education to inform future decision-making when taking up the appropriate practice. Recent scholarship has elucidated the many ways that using equity pedagogies in CS classrooms can positively influence student outcomes, such as achievement, belonging, and interest (Madkins, 2016; A. Martin et al., 2017; Ryoo et al., 2013; K. Scott & White, 2013; Vakil, 2014). Other scholars have found the use of culturally relevant and responsive teaching to support students in making connections between social justice issues, CS course content, and how CS course content is relevant to their personal lives (Madkins, 2016; Madkins et al., 2019; A. Martin et al., 2017; K. Scott et al., 2015).

Scott and White (2013) utilized culturally relevant computing (CRC) principles in their curriculum for COMPUGIRLS, a two-year program for girls, mostly from minoritized communities (74% Latin@, 19% African American). From 2009 – 2011, 41 adolescent girls (mean age = 14.5 years) engaged in learning tech-focused content and social justice issues, yielding increased identification with and interest in the tech fields. The use of CRC practices facilitated girls’ increased tech knowledge, agency, and critical consciousness development. Vakil (2014) highlights the importance of connecting app programming to critical consciousness development—a key component of equity-focused pedagogies. In his study of four focal high school students from minoritized communities in an after-school program in the Bay Area, students developed critical consciousness concurrently with computational literacies. Students expressed that having opportunities to make connections to social justice issues contributed to their increased interest, engagement, and participation in CS (Vakil, 2014).

A popular curriculum and course in public schools, Exploring Computer Science (ECS), was developed in 2008 to democratize CS learning in secondary classrooms through the use of culturally relevant curricula (Goode & Margolis, 2011; Margolis et al., 2012; Ryoo, 2019; Ryoo et al., 2013). ECS is grounded in students’ inquiry-based explorations of CS concepts and issues of equity and has been shown to be aligned with CRC principles (Ryoo, 2019). Over the course of a 3-year period, the number of students enrolled in ECS increased by 53% per year with a dramatic difference in participation rates of Black, Latinx, and girls in contrast to AP computer science (Ryoo et al., 2013). Across numerous classroom settings, using ECS has supported students in developing critical consciousness, seeing the relevance of CS to their lives, and learning CS concepts (Goode & Margolis, 2011; N. Howard, 2019; Madkins et al., 2019; Margolis et al., 2012; Ryoo, 2019; Ryoo et al., 2013).

## Practical Examples of Equity-Focused Teaching Practices in CS Learning Environments

Rather than providing an exhaustive and prescriptive list, in this section, we intend to share practical examples to demonstrate the possible ways educators and researchers might envision implementing equity-focused teaching in CS classrooms. We also recommend reviewing the *Resources for Further Learning* section of this manuscript, as well as prior work in the field towards this end (e.g., Madkins et al., 2019; Rankin & Thomas, 2020).

## Guidance for Educators and Researchers

When providing the background and context on the state of the field of computer science (CS), we noted the renewed interest in K-12 CS education over the last 15 years. From the Obama Administration announcement of the Computer Science for All initiative to the collective work informing the *K-12 Computer Science Framework* and subsequent standards, direct and indirect efforts are underway to improve the field and broaden participation in K-12 CS. An increased interest in broadening participation calls for initiatives aimed at relevant guidance on situating equity pedagogies as an essential practice that specifically consider minoritized students. When providing an overview of the state of the field, we also addressed the work of both ISTE and CSTA on sets of CS and CT standards for educators that address equity related to access and achievement. These sets of standards include words like *equity*, *inclusion*, and *culturally relevant learning*, demonstrating a recognition that there is still a need for the purposeful application of instructional practices. We commend the collective work of CTSA, ISTE, and other organizations. However, as critical scholars of STEM and CS education, we would be remiss if we did not acknowledge that what is written in a set of standards is often either not followed or explicitly followed absent of the critical consciousness or rationale required to effectively implement equity pedagogies.

In this article we not only provided a rationale for using equity pedagogies in computer science, we outlined practical examples from research on how practitioners might examine and select equity pedagogies for their classrooms. We specifically offered background and insights related to four equity-focused theoretical frameworks based on their grounding of assets-based approaches to supporting minoritized students as evidenced in prior research and practice. Although we differentiate these equity-focused theoretical frameworks, the application of each one positions educators to continually work towards centering equity in their teaching.

Effectively engaging equity pedagogies requires educators to support minoritized students by: 1) constructing culturally relevant and rigorous CS curricula; 2) implementing the curricula in a meaningful manner; 3) challenging systemic racism, power, and oppression; 4) guiding students in grappling with these same systems; and 5) encouraging them to use STEM and CS to empower themselves and their communities. When educators engage equity pedagogies students are supported in their identity development, understanding of the personal and sociopolitical relevance of technology, and positioned as creative agents and change agents.

For researchers, we contend that there is a need for more design-based research (K. Gutiérrez et al., 2017; Gutiérrez & Jurow, 2016; Vossoughi & K. Gutiérrez, 2014) and other modes of inquiry (i.e., qualitative research, mixed methods research) that consider the effectiveness of equity pedagogies in CS learning. We need varied approaches to conducting research in the field to provide a clearer picture of how equity-focused pedagogies in CS learning environments are taken up. Researchers must examine the range of in and out of school contexts where CS equity-focused teaching and learning occurs. In so doing, we can unpack the ways in which educators facilitate learning experiences and how—and to what extent—students learn CS content. Equity-focused research endeavors in STEM and CS are commonly critiqued for lacking ways to assess traditional student learning. Though we understand it is not always easy to do so due to a variety of reasons, including accessing sensitive student data, human subjects research approval, and long-term access to students in schools. Nonetheless, we encourage future research to explore multiple dimensions of student outcomes, including critical consciousness development, educational outcomes (i.e., interest, engagement, participation, etc.), and learning. This can be measured by conceptual understanding and distal measures of achievement, like grades or tests.

## Conclusion

In this position paper, we noted the importance of working towards equity in support of minoritized students. We specifically highlighted four assets-based equity pedagogies (CRP, CRT, CSP, and CRC) and posited that they should be taken up in K-12 computer science learning environments. We maintain there is a need to focus on the integration of equity pedagogies in K-12 CS learning environments and shift attention from broadening participation and achievement to directly supporting the needs of CS learners. In so doing, we further asserted that CRP, CRT, CSP, and CRC can and should be used in more than ELA and social studies. More specifically, equity pedagogies should be taken up in K-12 CS learning environments to support minoritized students.

STEM and computer science innovations remain an important factor in the growth and development of economies around the world. Minoritized students continue to contend with the denial of access to advanced courses, as well as rigorous instruction in STEM and CS instruction. Much attention has been given to broadening participation for minoritized students and there have been notable advances in CS educational research and practitioner communities related to equity-focused teaching. While we have noted such advancements in this article, it is important to recognize that there is more work to do to further support students with the integration of CS knowledge in K-12 learning environments. Such an integration can occur by prioritizing equity pedagogies in CS learning environments that provide access to instructional practices to effectively prepare students for entry into STEM and CS fields. Taken together, scholarly, practitioner, and professional computer science communities are increasingly focused on discussing and addressing issues of equity, equity-focused teaching strategies, and fostering more inclusive CS and tech spaces. It is our hope that this article further supports these collective efforts to support minoritized youth in further developing their CS knowledge, seeing the connections between using this knowledge and pursuing their personal interests, and using computational thinking to empower themselves and their communities.

## Figures and Tables

**Table 1:** Differentiating Equity-Focused Theoretical Frameworks

Theoretical Framework	Overview
*Culturally Relevant Pedagogy* (CRP) Ladson-Billings (1995, 2006)	Three tenets include educators’ intentional mindset and explicit actions related to: academic excellence (*explicit* high expectations for student learning and achievement); cultural competence (using students’ cultural practices for learning and breaking down dominant culture in schooling); and critical/sociopolitical consciousness development (awareness of and challenging of sociopolitical forces influencing our world).
*Culturally Responsive Teaching* (CRT) Gay (2000) Hammond (2015a/b)	The five essential elements of culturally responsive teaching include: cultural diversity knowledge base; culturally relevant curricula; cultural care and learning communities; cross-cultural communications; and cultural congruity.
*Culturally Sustaining Pedagogy* (CSP) Paris & Alim (2012, 2014, 2017)	Focus on maintaining and cultivating linguistic and cultural pluralism, in opposition to existing educational contexts historically structured to ignore and marginalize students’ cultural and linguistic assets.
*Culturally Responsive Computing* (CRC) K. Scott et al. (2013, 2015)	Draws and builds upon principles of culturally relevant teaching to honor student backgrounds, life experiences, and interests to make meaningful connections to computing topics.

**Table 2:** Engaging Equity-Focused Teaching Practices in Learning Environments

Identity development	Are aware of and address power dynamics that impact classroom culture and student experience. For example, educators are aware of racialized, gendered, and other stereotypes that may influence how students see themselves and others. In turn, educators implicitly and explicitly position all students, especially minoritized students, as capable learners within the learning environment. Are aware that intersectional identities can impact student experiences with technology in different ways and on different levels. This might include how the effects of technology impact different communities and populations differently, identity impacts on access to technology/technology learning, identity impacts on ability to see oneself as a technological change agent. For example, educators should be aware of gendered disparities in CS and tech fields with their students and how these are exacerbated along racialized, classed/socioeconomic, and other sociocultural identity markers. Support student identity development and expression through computing tools. For example, educators should give students enhanced control over presentations of self in computing projects and the selection of computing tools. Challenge stereotypes about who belongs in computing by addressing representation, showing students examples of diverse creators in STEM and creating space for discussion. Encourage peer pedagogy, where students teach and learn from each other via feedback and sharing ideas/completed work (for a full explanation, see Ching & Kafai, 2008 or Fields et al., 2018). Support student friendships.
Personal and sociopolitical relevance of technology	Situate technology ideas within their sociopolitical context and give students opportunities to critique and explore issues that are relevant to them. For example, educators might invite students to participate in discussions about how and why Internet access is inequitable in the U.S., how particular technologies have been used to racially profile certain populations, or current events related to tech tools. Create CS experiences that connect to student knowledge, interests, and life experiences, broadening ideas of where CS thinking can be applied. For example, educators may have students develop an app that connects CS knowledge and skills to sociopolitical issues relevant to students and their communities. They can provide examples, like the app developed by three teenaged siblings from Georgia to document police brutality in their neighborhoods. (See Borison, 2014 for details about the app.) Value students’ strengths and outside knowledge and create opportunities for them to showcase these.
Positioning students as creative agents/change agents	Empower learners to become creative innovators with technology, able to repurpose technology towards their own goals. Build enough flexibility into both the tools and the activities to leave room for students to pursue goals that are meaningful to them. Legitimize student expertise through creating opportunities to share their work with the broader community and/or making their work visible in a shared space.

**Sources:** Ashcraft et al. (2017); Babbitt et al. (2012); Fields et al. (2018); Keune et al. (2019); Pinkard et al. (2017); Ryoo (2019); Scott et al. (2015); Vakil (2018)

## Resources for Further Learning

As discussed in this article, using equity pedagogies with fidelity requires teachers to simultaneously focus on learning *and* critical consciousness development. Review this list of resources for further learning related to researching and using equity pedagogies in CS learning environments.

- **Read this book and view the accompanying website** if you work in K-5 learning environments: Howard, N. R., & Howard, K. E. (2020). *Coding + math: Strengthen K–5 math skills with computer science*. International Society for Technology in Education. Check out the accompanying website: https://www.k12stemequity.com/
- **Learn** from Shana V. White, an extraordinary CS educator in suburban Atlanta, Georgia: https://shanavwhite.com/.
- **Read this research paper and view this presentation** to learn more about the distinctions between race, power, justice, and related terms as we focus on equity in CS education: Lewis, C. M., Goode, J., Scott, A., Shah, N., & Vakil, S. (2020). Researching race in computer science education: Demystifying key vocabulary and methods. Paper presented at the Proceedings of the 51st ACM Technical Symposium on Computer Science Education. 10.1145/3328778.3366965
- **Read and use** this text and the accompanying website for K-12 learning environments: Grover, S. (2020). *Computer science in K-12: An A-to-Z handbook on teaching programming*. Looking Glass Ventures. https://www.shuchigrover.com/atozk12cs/
- **Review and use** this online repository developed by Tia C. Madkins and her research team (2017) for specific suggestions for how to connect secondary CS content to social justice issues.
- **Review and use** these resources for social justice-focused STEM teaching curated by Dr. Kari Kokka
- **Read** this report to better understand why and how we might teach CS with issues of diversity and equity in mind: Santo, R., Vogel, S., & Ching, D. (2019). *CS for What? Diverse Visions of Computer Science Education in Practice*.
- **Review and use** this resource portal if planning a research project focused on broadening participation in CS, especially for NSF-sponsored projects.

## Footnotes

1. See Patton Davis & Museus (2019) for a review of deficit-based approaches and thinking, which is viewing students, families, and communities’ ways of knowing and doing as deficient or in need of repair/fixing. This is in contrast to assets-based approaches that view students, families, and communities’ practices as assets that educators might use for learning; see this post (https://teachereducation.steinhardt.nyu.edu/an-asset-based-approach-to-education-what-it-is-and-why-it-matters/) for a more detailed description.

2. We purposefully state that students can “further develop their potential to become social change agents” because we do not believe students come to our classrooms without already having developed their potential to be social change agents. Rather, many of our students, especially students from minoritized communities who experience intersecting forms of oppression, are already developing or doing this.

3. See Ngo et al. (2017) for a review of the dimensions of sociopolitical consciousness or Diemer et al. (2017) for research related to a scale for measuring sociopolitical consciousness.

## References

[R1] Abt-Perkins D. (2011). Foreword. Looking for a little inspiration. In Scherff L & Spector K (Eds.), Culturally relevant pedagogy: Clashes and confrontations (pp. v–ix). Rowman and Littlefield Education Publishers, Inc.

[R2] Akhtar A. (2019, October 28). New York is investigating UnitedHealth's use of a medical algorithm that steered Black patients away from getting higher-quality care. Business Insider.com. https://www.businessinsider.com/an-algorithm-treatment-to-white-patients-over-sicker-black-ones-2019-10

[R3] Allen KM, Jackson I, & Knight MG (2012). Complicating culturally relevant pedagogy: Unpacking African immigrants' cultural identities. International Journal of Multicultural Education, 14(2). 10.18251/ijme.v14i2.506

[R4] Apple and ConnectED Initiative. https://www.apple.com/connectED/

[R5] Aronson B, & Laughter J (2016). The theory and practice of culturally relevant education: A synthesis of research across content areas. Review of Educational Research, 86, 163–206. 10.3102/0034654315582066

[R6] Ashcraft C, Eger EK, & Scott KA (2017). Becoming technosocial change agents: Intersectionality and culturally responsive pedagogies as vital resources for increasing girls’ participation in computing. Anthropology & Education Quarterly, 48, 233–251. 10.1111/aeq.12197

[R7] Babbitt B, Lyles D, & Eglash R (2012). From ethnomathematics to ethnocomputing: Indigenous algorithms in traditional context and contemporary simulation. In Mukhopadhyay S, & Roth W-M (Eds.), Alternative forms of knowing (in) mathematics (pp. 205–219). Brill Sense. 10.1007/978-94-6091-921-3_10

[R8] Bang M. (2020). Learning on the move toward just, sustainable, and culturally thriving futures. Cognition and Instruction, 38, 434–444. 10.1080/07370008.2020.1777999

[R9] Banks JA (2006). Race, culture, and education: The selected works of James A. Banks. Routledge.

[R10] Banks CAM & Banks JA (1995). Equity pedagogy: An essential component of multicultural education. Theory into Practice, 34, 151–158. 10.1080/00405849509543674

[R11] Benjamin R. (2016). Catching our breath: Critical race STS and the carceral imagination. Engaging Science, Technology, and Society, 2, 145–156. 10.17351/ests2016.70

[R12] Benjamin R. (2019). Race after technology: Abolitionist tools for the New Jim Code. Polity. Black Girls Code. https://www.blackgirlscode.com/

[R13] Borison R. (2014, August 18). Youngsters create an app to document police abuse. Slate.com https://slate.com/business/2014/08/three-teens-create-five-o-an-app-to-document-police-abuse-in-the-wake-of-ferguson-riots.html

[R14] Brannon M, & Novak E (2019). Coding success through math intervention in an elementary school in rural Amish country. Journal of Computer Science Integration, 2(2), 1–10. 10.26716/jcsi.2019.02.2.1

[R15] Brathwaite LF (2020, August 21). Why dating apps are racist AF—With or without ethnicity filters. RollingStone.com https://www.rollingstone.com/culture/culture-features/dating-apps-grindr-ethnicity-filters-1047047/

[R16] Bryan LA, & Atwater MM (2002). Teacher beliefs and cultural models: A challenge for science teacher preparation programs. Science Education, 86, 821–839. 10.1002/sce.10043

[R17] Buranyi S. (2017, August 8). https://www.theguardian.com/inequality/2017/aug/08/rise-of-the-racist-robots-how-ai-is-learning-all-our-worst-impulses

[R18] Caeli EN, & Yadav A (2020). Unplugged Approaches to Computational Thinking: a Historical Perspective. TechTrends, 64(1), 29–36. 10.1007/s11528-019-00410-5

[R19] Calabrese Barton A (2003). Teaching science for social justice. Teachers College Press.

[R20] Carter PL, & Welner KG (2013). Closing the opportunity gap: What America must do to give every child an even chance. Oxford University Press. 10.1093/acprof:oso/9780199982981.001.0001

[R21] Chakrabarti M, Carter PL, Kendi I (2019, September 9). Part I: Achievement gap, or opportunity gap? What’s stopping student success. WBUR Closing the achievement gap series. https://www.wbur.org/onpoint/2019/09/09/achievement-gap-opportunity-education-schools-students-teachers

[R22] Cheney-Lippold J. (2017). We are data: Algorithms and the making of our digital selves. New York University Press. 10.2307/j.ctt1gk0941

[R23] Ching CC, & Kafai YB (2008). Peer pedagogy: Student collaboration and reflection in a learning-through-design project. Teachers College Record, 110, 2601–2632.

[R24] Cochran-Smith M. (2003). Standing at the crossroads: Multicultural teacher education at the beginning of the 21st century. Multicultural Perspectives, 5(3), 3–11. 10.1207/s15327892mcp0503_02

[R25] Code.org. (2019). 2019 State of Computer Science Education. https://advocacy.code.org/2019_state_of_cs.pdf

[R137] Code Mississippi. http://www.codems.net/

[R26] Computer Science for ALL Students. (CSforAll). (2020). https://www.csforall.org/

[R27] CSforAll. (2020). Equitable CS district learning cohort. https://www.csforall.org/projects_and_programs/equitable-cs-district-learning-cohort/

[R28] Computer Science Teachers Association (CSTA). (2020). CSTA K-12 CS Standards. https://www.csteachers.org/page/standards

[R29] CSTA Equity Fellows. https://csteachers.org/Stories/csta-announces-the-2020%E2%80%9321-equity-fellowship-cohort

[R30] Cummings R, Chambers B, Reid A, & Gosha K (2019, April). STEM hip-hop pedagogy: A meta-synthesis on hip-hop pedagogy STEM interventions tools for underrepresented minorities in K-12 education. In Proceedings of the 2019 Association for Computing Machinery (ACM) Southeast Conference (pp. 46–52). 10.1145/3299815.3314431

[R31] Cuny J. (2012). Transforming high school computing: A call to action. ACM Inroads 3, (2), 32–36. 10.1145/2189835.2189848

[R32] Dell Foundation. Classroom supports. https://www.dell.org/what-we-do/classroom-supports/

[R33] desJardins M. (2015). Creating AP^®^ CS principles: Let many flowers bloom. ACM Inroads, 6(4), 60–66. 10.1145/2835852

[R34] Diemer MA, Rapa LJ, Park CJ, & Perry JC (2017). Development and validation of the Critical Consciousness Scale. Youth & Society, 49, 461–483. 10.1177/0044118x14538289

[R35] Diente J. (2020, September 25). Gradient app draws criticism over AI Face feature which purportedly promotes digital ‘blackface’. MSN.com

[R36] Espinoza ML, Vossoughi S, Rose M, & Poza LE (2020). Matters of participation: Notes on the study of dignity and learning. Mind, Culture, and Activity, 1–23. 10.1080/10749039.2020.1779304

[R37] Espinoza O. (2007). Solving the equity–equality conceptual dilemma: A new model for analysis of the educational process. Educational Research, 49, 343–363. 10.1080/00131880701717198

[R38] Exploring Computer Science (ECS). http://www.exploringcs.org/

[R39] Fields DA, Kafai Y, Nakajima T, Goode J, & Margolis J (2018). Putting making into high school computer science classrooms: Promoting equity in teaching and learning with electronic textiles in Exploring Computer Science. Equity & Excellence in Education, 51, 21–35. 10.1080/10665684.2018.1436998

[R40] Freire P. (1970/2005). Pedagogy of the oppressed. Continuum.

[R41] Garcia A, & Morrell E (2013). City youth and the pedagogy of participatory media. Learning, Media and Technology, 38, 123–127. 10.1080/17439884.2013.782040

[R42] Gaskins N. (2016, July 26). How art and dance are making computer science culturally relevant. EdSurge News. https://www.edsurge.com/news/2016-07-26-how-art-and-science-are-making-computer-science-culturally-relevant

[R43] Gay G. (2000). Culturally responsive teaching: Theory, research, and practice. Teachers College Press.

[R44] Gay G, & Kirkland K (2003). Developing cultural critical consciousness and self-reflection in preservice teacher education. Theory Into Practice, 42, 181–187. 10.1207/sl5430421tip42033

[R45] Gholson M, & Martin DB (2014). Smart girls, Black girls, mean girls, and bullies: At the intersection of identities and the mediating role of young girls' social network in mathematical communities of practice. Journal of Education, 194, 19–33. 10.1177/002205741419400105

[R46] Gholson M, & Martin DB (2019). Blackgirl face: Racialized and gendered performativity in mathematical contexts. ZDM Mathematics Education, 51, 391–404. 10.1007/s11858-019-01051-x

[R47] Gilpin L. (2015, February 4). Diversity in tech: 10 data points you should know. Tech Republic. https://www.techrepublic.com/article/diversity-in-tech-10-data-points-you-should-know/

[R48] Goode J, & Margolis J (2011). Exploring Computer Science: A case study of school reform. ACM Transactions on Computing Education, 11(2), Article 12, 1–16. 10.1145/1993069.1993076

[R49] Goode J, Ivey A, RunningHawk Johnson S, Ryoo JJ, & Ong C (2020). Rac(e)ing to computer science for all: How teachers talk and learn about equity in professional development. Computer Science Education, Advance Online Release, 1–26. 10.1080/08993408.2020.1804772

[R50] Google Education. Helping expand learning for everyone. https://edu.google.com/why-google/our-commitment/?modal_active=none

[R51] Gutiérrez KD, & Jurow AS (2016). Social design experiments: Toward equity by design. Journal of the Learning Sciences, 25, 565–598. 10.1080/10508406.2016.1204548

[R52] Gutiérrez KD, Cortes K, Cortez A, DiGiacomo D, Higgs J, Johnson P, Lizárraga JR, Mendoza E, Tien J, & Vakil S (2017). Replacing representation with imagination: Finding ingenuity in everyday practices. Review of Research in Education, 41, 30–60. 10.3102/0091732x16687523

[R53] Gutiérrez R. (2002). Enabling the practice of mathematics teachers in context: Toward a new equity research agenda. Mathematical Thinking and Learning, 4, 145–187. 10.1207/s15327833mtl04023_4

[R54] Gutiérrez R (2008). Framing equity: Helping students “play the game” and “change the game.” Noticias de Todos/TODOS: Mathematics for ALL, 4(1), 1–3.

[R55] Guynn J. (2015, July 1). Google Photos labeled black people 'gorillas.' USA Today. https://www.usatoday.com/story/tech/2015/07/01/google-apologizes-after-photos-identify-black-people-as-gorillas/29567465/

[R56] Guzdial M. (2020, June 5). CS teachers, It's (past) time to learn about race. Blog@CACM. https://cacm.acm.org/blogs/blog-cacm/245408-cs-teachers-its-past-time-to-learn-about-race/fulltext

[R57] Guzdial M. (2020, June 8). Becoming anti-racist: Learning about race in CS Education. Computing Education Research Blog. https://computinged.wordpress.com/2020/06/08/lets-talk-about-race-in-cs-education-more-resources/

[R58] Hammond Z. (2015a, April 9). Four tools for interrupting implicit bias. Culturally Responsive Teaching and the Brain. https://crtandthebrain.com/four-tools-for-interrupting-implicit-bias/

[R59] Hammond Z. (2015b). Culturally Responsive Teaching and the Brain: Promoting authentic engagement and rigor among culturally and linguistically diverse students. Corwin: A Sage Company.

[R60] Heitin L. (2015). The state of computer science in U.S. high schools: An administrator’s perspective. Education Week, 34(17), 5.

[R61] Hines SM (Ed.) Multicultural science education: Theory, practice, and promise. Peter Lang Publishing.

[R62] Howard KE, & Havard DD (2019). Advanced Placement (AP) Computer Science Principles: Searching for equity in a two-tiered solution to underrepresentation. Journal of Computer Science Integration, 2(1), 1–15. 10.26716/jcsi.2019.01.1.1

[R63] Howard NR (2019). EdTech Leaders’ Beliefs: How are K-5 teachers supported with the integration of computer science in K-5 classrooms?. Technology, Knowledge and Learning, 24(2), 203–217. 10.1007/s10758-018-9371-2

[R64] Howard NR & Howard KE (2020). Coding+Math: Strengthen K-5 Math Skills with Computer Science. Portland, OR: International Society for Technology in Education.

[R65] Howard TC (2001). Powerful pedagogy for African American students: A case of four teachers. Urban Education, 36, 179–202. 10.1177/0042085901362003

[R66] Howard TC (2003). Culturally relevant pedagogy: Ingredients for critical teacher reflection. Theory Into Practice, 42, 195–202. 10.1207/s15430421tip4203_5

[R67] Howard TC (2012). Culturally responsive pedagogy. In Banks JA (Ed.), Encyclopedia of diversity in education (pp. 549–552). Sage Publications.

[R68] IBM Quantum Education and Research Initiative for Historically Black Colleges and Universities. https://newsroom.ibm.com/2020-09-17-IBM-Establishes-First-Quantum-Education-and-Research-Initiative-for-Historically-Black-Colleges-and-Universities

[R69] International Society for Technology in Education (ISTE). (2016/2017). The ISTE Standards. https://www.iste.org/standards

[R70] Jacob SR, & Warschauer M (2018). Computational Thinking and Literacy. Journal of Computer Science Integration, 1(1). 10.26716/jcsi.2018.01.1.1

[R71] Keune A, Peppler KA, & Wohlwend KE (2019). Recognition in makerspaces: Supporting opportunities for women to “make” a STEM career. Computers in Human Behavior, 99, 368–380. 10.1016/j.chb.2019.05.013

[R72] Koeze E, & Popper N (2020, April 7). The virus changed the way we Internet. New York Times. https://www.nytimes.com/interactive/2020/04/07/technology/coronavirus-internet-use.html

[R73] Ladson-Billings G. (1995). But that's just good teaching! The case for culturally relevant pedagogy. Theory into practice, 34, 159–165. 10.1080/00405849509543675

[R74] Ladson-Billings G. (2006). “Yes, but how do we do it?” Practicing culturally relevant pedagogy. In Landsman J & Lewis CW (Eds)., White teachers/diverse classrooms: A guide to building inclusive schools, promoting high expectations, and eliminating racism (pp. 29–42). Stylus Publishing, LLC. 10.1080/00131940701634718

[R75] Ladson-Billings G. (2012, April). Culturally relevant pedagogy as an “equity pedagogy.” In Banks JA (Chair), Presidential Session. Symposium conducted at the meeting of American Educational Research Association, Vancouver, British Columbia. 10.3102/00028312032003465

[R76] Lee CD (2017). An ecological framework for enacting culturally sustaining pedagogy. In Paris D & Alim HS (Eds.), Culturally sustaining pedagogies: Teaching and learning for justice in a changing world, (pp. 261–274). Teachers College Press.

[R77] Lee CH, & Soep E (2016). None but ourselves can free our minds: Critical computational literacy as a pedagogy of resistance. Equity & Excellence in Education, 49, 480–492. 10.1080/10665684.2016.1227157

[R78] Leonard J, & Martin DB (2013). The brilliance of Black children in mathematics. Information Age Publishing.

[R79] Madkins TC (2016). Empowering Teachers to Change: A Mixed Methods Examination of Equity-Oriented STEM Instruction. Dissertation, eScholarship, University of California, Berkeley.

[R80] Madkins TC, Martin A, Ryoo J, Scott KA, Goode J, Scott A, & McAlear F (2019). Culturally relevant computer science pedagogy: From theory to practice. 2019 Research on Equity and Sustained Participation in Engineering, Computing, and Technology (RESPECT) Conference Proceedings, Minneapolis, MN, USA, (pp. 1–4). 10.1109/respect46404.2019.8985773

[R81] Madkins TC, Thomas JO, Solyom J, Goode J, & McAlear F (2020). Learner-centered and culturally relevant pedagogy. In Grover S (Ed.), Computer science in K-12: An A-to-Z handbook on teaching programming (pp. 125–129). Looking Glass Ventures.

[R82] Margolis J, Estrella R, Goode J, Holme J, & Nao K (2008). Stuck in the shallow end: Education, race, and computing. MIT Press. 10.7202/1003580ar

[R83] Margolis J, & Goode J (2016). Ten lessons for CS for All. ACM Inroads, 7(4), 52–56. 10.1145/2988236

[R84] Margolis J, Ryoo JJ, Sandoval CDM, Lee C, Goode J, Chapman G (2012). Beyond Access: Broadening participation in high school computer science. ACM Inroads 3(4), 72–78. 10.1145/2381083.2381102

[R85] Martin A, Madkins TC, & McAlear F (2017, April). Leveling the coding field: Culturally relevant computer science in the SMASH Academy. Paper presented at the annual meeting of the American Educational Research Association, San Antonio, TX.

[R86] Martin DB (2003). Hidden assumptions and unaddressed questions in *Mathematics for All* rhetoric. The Mathematics Educator, 13(2), 7–21. https://openjournals.libs.uga.edu/tme/article/view/1856

[R87] Martin DB (2009). Researching race in mathematics education. Teachers College Record, 111, 295–338.

[R88] Microsoft. (2019). Guide to inclusive computer science education: How educators can encourage and engage all students in computer science. https://csteachers.org/documents/en-us/2730df36-1cb1-422b-ba2f-afe033750b2e/1/

[R89] Mensah FM, & Larson K (2017). A summary of inclusive pedagogies for science education. [Background paper for the Board on Science Education of the National Academy of Sciences, Engineering, and Medicine]. Department of Mathematics, Science & Technology, Teachers College, Columbia University.

[R90] Milner HR (2011). Culturally relevant pedagogy in a diverse urban classroom. Urban Review, 43, 66–89. 10.1007/s11256-009-0143-0

[R91] Mitchum R. (2020, July 17). How computer science can help fight COVID-19. University of Chicago News. https://news.uchicago.edu/story/how-computer-science-can-help-fight-covid-19

[R92] Morales-Chicas J, Castillo M, Bernal I, Ramos P, & Guzman BL (2019). Computing with relevance and purpose: A review of culturally relevant education in computing. International Journal of Multicultural Education, 21, 125–155. 10.18251/ijme.v21i1.1745

[R93] Morales-Doyle D, Vossoughi S, Vakil S, & Bang M (2020, August 19). In an era of pandemic, STEM education can’t pretend to be apolitical. Truthout Education and Youth Op-Ed. https://truthout.org/articles/in-an-era-of-pandemic-and-protest-stem-education-cant-pretend-to-be-apolitical/?utm_campaign=Truthout+Share+Buttons

[R94] National Assessment of Educational Progress (NAEP). (2016). Scores 2015. https://www.nationsreportcard.gov/science_2015/

[R95] National Research Council (NRC). (2012). A framework for K-12 education: Practices, crosscutting concepts, and ideas. National Academies Press.

[R96] National Science Board (NSB). (2020). The state of U.S. science and engineering 2020. National Science Foundation. https://ncses.nsf.gov/pubs/nsb20201

[R97] National Science Foundation (NSF). Broadening Participation in Computing Alliance Program. https://www.nsf.gov/funding/pgm_summ.jsp?pims_id=503593

[R98] NSF. NSF HBCU Undergraduate Program. https://www.nsf.gov/funding/pgm_summ.jsp?pims_id=5481

[R99] Ngo B, Lewis C, & Maloney Leaf B (2017). Fostering sociopolitical consciousness with minoritized youth: Insights from community-based arts programs. Review of Research in Education, 41, 10.3102/0091732x17690122

[R100] Ong M, Wright C, Espinosa L, & Orfield G (2011). Inside the double bind: A synthesis of empirical research on undergraduate and graduate women of color in science, technology, engineering, and mathematics. Harvard Educational Review, 81, 172–209. 10.17763/haer.81.2.t022245n7x4752v2

[R101] Ortiz NA, Morton TR, Miles ML, & Roby RS (2020). What about us? Exploring the challenges and sources of support influencing Black students’ STEM identity development in postsecondary education. The Journal of Negro Education, 88, 311–326. 10.7709/jnegroeducation.88.3.0311

[R102] Papert S. (1980, 1993). Mindstorms. Children, computers, and powerful ideas. Basic Books

[R103] Paris D. (2012). Culturally sustaining pedagogy: A needed change in stance, terminology, and practice. Educational researcher, 41(3), 93–97. 10.3102/0013189x12441244

[R104] Paris D, & Alim HS (2014). What are we seeking to sustain through culturally sustaining pedagogy? A loving critique forward. Harvard Educational Review, 84, 85–100. 10.17763/haer.84.1.982l873k2ht16m77

[R105] Paris D, & Alim HS (Eds.) (2017). Culturally sustaining pedagogies: Teaching and learning for justice in a changing world. Teachers College Press.

[R106] Patton Davis L, & Museus SD (2019, July 19). Identifying and disrupting deficit thinking. Medium.com. https://medium.com/national-center-for-institutional-diversity/identifying-and-disrupting-deficit-thinking-cbc6da326995

[R107] Payton, (2020). Jamie Payton and Tiffany Barnes, RESPECT Steering Committee Co-Chairs Christina Gardner-McCune and Nicki Washington, N. (2020). STCBP & RESPECT statement on addressing racism & injustice. http://stcbp.org/respect-statement-on-addressing-racism-injustice/

[R108] Pinkard N, Erete S, Martin CK, & McKinney de Royston M (2017). Digital youth divas: Exploring narrative-driven curriculum to spark middle school girls’ interest in computational activities. Journal of the Learning Sciences, 26, 477–516. 10.1080/10508406.2017.1307199

[R109] Pinkerton B. (2016, August 12). He’s brilliant, she’s lovely: Teaching computers to be less sexist. NPR. https://www.npr.org/sections/alltechconsidered/2016/08/12/489507182/hes-brilliant-shes-lovely-teaching-computers-to-be-less-sexist

[R110] Rankin, & Thomas JO (2020). The intersectional experiences of Black women in computing. In Proceedings of the 51st ACM Technical Symposium on Computer Science Education (pp. 199–205). 10.1145/3328778.3366873

[R111] Resnick B. (2019, January 24). Yes, artificial intelligence can be racist. Vox.com

[R112] Research in Equity and Sustained Participation in Engineering, Computing, and Technology (RESPECT). http://respect2021.stcbp.org/

[R113] Ryoo JJ, Margolis J, Lee CH, Sandoval CDM, & Goode J (2013). Democratizing computer science knowledge: Transforming the face of computer science through public high school education. Learning, Media, and Technology, 1–21. 10.1080/17439884.2013.756514

[R114] Ryoo JJ (2019). Pedagogy that supports Computer Science for All. ACM Transactions on Computing Education 19(4), Article 36, 1–23. 10.1145/3322210

[R115] Sargrad S, Harris KM, Partelow L, Campbell N, & Jimenez L (2019, July 2). A quality education for every child: A new agenda for education policy. Center for American Progress. https://www.americanprogress.org/issues/education-k-12/reports/2019/07/02/471511/quality-education-every-child/

[R116] Scott A, Martin A, McAlear F, & Madkins TC (2016, December). Broadening participation in computer science: Existing out-of-school initiatives and a case study. ACM Inroads, 7, 84–90. 10.1145/2994153

[R117] Scott A, Klein FK, & Onovakpuri U (2017). Tech leavers study. Kapor Center. https://www.kaporcenter.org/tech-leavers/

[R118] Scott KA, & Clark K (2013). Digital engagement for urban youth: From theory to practice. Urban Education, 48, 627–628. 10.1177/0042085913490556

[R119] Scott KA, & White M (2013). COMPUGIRLS’ Standpoint: Culturally responsive computing and its effect on girls of color. Urban Education, 48, 657 – 681. 10.1177/0042085913491219

[R120] Scott KA, Sheridan KM, & Clark K (2015). Culturally responsive computing: A theory revisited. Learning, Media and Technology, 40, 412–436. 10.1080/17439884.2014.924966

[R121] Secada WG (1989). Agenda setting, enlightened self-interest, and equity in mathematics education. Peabody Journal of Education, 66(2), 22–56. 10.1080/01619568909538637

[R122] Sengupta-Irving T, & Vossoughi S (2019). Not in their name: Re-interpreting discourses of STEM learning through the subjective experiences of minoritized girls. Race Ethnicity and Education, 22, 479–501. 10.1080/13613324.2019.1592835

[R123] Sherriff M, Merkle L, Cutter P, Monge A, & Sheard J (2020). Statement on equity. Association for Computing Machinery (ACM) Special Interest Group on Computer Science Education (SIGCSE) Technical Symposium and Program Co-Chairs. 10.1145/3419040.3419044

[R124] Sleeter CE (2012). Confronting the marginalization of culturally responsive pedagogy. Urban Education, 20, 1–23. 10.1177/0042085911431472

[R125] Smith M. (2016, January 30). Computer science for all summary. The White House (President Barack Obama). https://obamawhitehouse.archives.gov/blog/2016/01/30/computer-science-all

[R126] Tate WF (1995). Returning to the root: A culturally relevant approach to mathematics pedagogy. Theory Into Practice, 34, 166–173. 10.1080/00405849509543676

[R127] Tozzi C. (2020, September 3). Cloud computing trends during the COVID-19 pandemic. ITProToday. https://www.itprotoday.com/hybrid-cloud/cloud-computing-trends-during-covid-19-pandemic

[R128] Vakil S. (2014). A critical pedagogy approach for engaging urban youth in mobile app development in an after-school program. Equity and Excellence in Education, 47, 31–45. 10.1080/10665684.2014.866869

[R129] Vakil S. (2018). Ethics, identity, and political vision: Toward a justice-centered approach to equity in computer science education. Harvard Educational Review, 88, 26–52. 10.17763/1943-5045-88.1.26

[R130] Vakil S, & Ayers R (2019). The racial politics of STEM education in the USA: Interrogations and explorations. Race Ethnicity and Education, 22, 449–458. 10.1080/13613324.2019.1592831

[R131] Vossoughi S, & Gutiérrez KD (2014). Studying movement, hybridity, and change: Toward a multi-sited sensibility for research on learning across contexts and borders. National Society for the Study of Education, 113, 603–632.

[R132] Watkins SE, & Mensah FM (2019). Peer support and STEM success for one African American female engineer. The Journal of Negro Education, 88, 181–193. 10.7709/jnegroeducation.88.2.0181

[R133] White SV (2017, July 9). Why do I cause you discomfort? Blog post. https://shanavwhite.com/2017/07/09/why-do-i-cause-you-discomfort/

[R134] Williams J, Carter PL, & Rearson S (2014). Researchers on opportunity gap, student inputs, outputs. Podcast on bloomberg.com. https://ed.stanford.edu/in-the-media/researchers-opportunity-gap-student-inputs-outputs-featuring-prudence-carter-and-sean

[R135] Wilson C, Sudol LA, Stephenson C, & Stehlik M (2010). Running on empty: The failure to teach K-12 computer science in the digital age. Executive Summary. Association for Computing Machinery, 1–13. 10.1145/3414583

[R136] Young E. (2010). Challenges to conceptualizing and actualizing culturally relevant pedagogy: How viable is the theory in classroom practice? Journal of Teacher Education, 61, 248–260. 10.1177/0022487109359775

